# SWPER Global: A survey-based women's empowerment index expanded from Africa to all low- and middle-income countries

**DOI:** 10.7189/jogh.10.020434

**Published:** 2020-12

**Authors:** Fernanda Ewerling, Anita Raj, Cesar G Victora, Franciele Hellwig, Carolina VN Coll, Aluisio JD Barros

**Affiliations:** 1 International Center for Equity in Health, Federal University of Pelotas, Pelotas, Brazil; 2 Postgraduate Program in Epidemiology, Federal University of Pelotas, Pelotas, Brazil; 3 Center on Gender Equity and Health, University of California San Diego, California, USA

## Abstract

B**ackground** In 2017, a survey-based women’s empowerment index (SWPER) was proposed for African countries, including three domains: social independence, decision making and attitude to violence. External validity and predictive value of the SWPER has been demonstrated in terms of coverage of maternal and child interventions and use of modern contraception. To determine its value for global monitoring, we explored the applicability of the SWPER in national health surveys from low- and middle- income countries (LMICs) in other world regions.

**Methods:**

We used data from the latest Demographic and Health Survey for 62 LMICs since 2000. 14 pre-selected questions (items) were considered during the validation process. Content adaptations included the exclusion of women’s working status and recategorization of the decision-making related items. We compared the loading patterns obtained from principal components analysis performed for each country separately with those obtained in a pooled data set with all countries combined. Country rankings based on the score of each SWPER domain were correlated with their rankings in the Gender Development Index (GDI) and the Gender Inequality Index (GII) for external validation.

**Results:**

Consistency regarding item loadings for the three SWPER empowerment domains was observed for most countries. Correlations between the scores generated for each country and global score obtained from the combined data were 0.89 or higher for all countries. Correlations between the country rankings according to SWPER and GDI were, respectively, 0.74, 0.72 and 0.67 for social independence, decision-making, and attitude to violence domains. The correlations were equal to 0.81, 0.67, and 0.44, respectively, with GII.

**Conclusions:**

The indicator we propose, named SWPER Global, is a suitable common measure of women’s empowerment for LMICs, addressing the need for a single consistent survey-based indicator of women´s empowerment that allows for tracking of progress over time and across countries at the individual and country levels.

Empowerment is a complex and multidimensional construct, often defined both as a process and an outcome, by which individuals gain power over their lives and decisions [1]. To be empowered, women must not only have key assets (such as education and health) and access to opportunities (such as employment), but also have the agency (ie, perceived and actual self-efficacy and decision-making control) to move from making planned choices to achieving one’s self-determined goals [2,3]. At the individual level, empowerment involves women utilizing their assets, opportunities, and agency for making purposive choices and engaging in behaviors to alter life circumstances. This may include engagement in positive behaviors and action towards health and economic development [4-6]. The Millennium Development Goals raised the women’s empowerment agenda in the recent past, by recognizing its importance for health as well as development, yet gender inequalities persist [7,8]. Women’s Human Development Index is, on average, 6% lower than that of men, with the widest gaps observed in the poorest countries. Much of the gap is due to women’s lower educational attainment, economic participation and income [7,9]. In 2015, the fifth Sustainable Development Goal (SDG) called for “achievement of gender equality and the empowerment of all women and girls” as a vital goal for accelerating sustainable development. However, there is no consensus on how to measure women’s empowerment and an internationally standardized l indicator is still unavailable, which precludes accountability in low-and middle-income countries (LMICs) [10].

In the recent years, some measures that capture gender inequalities in key socioeconomic and health indicators (eg, Gender Development Index, Global Gender Gap Index [7]), gender discrimination and gender-related risks for women such as maternal mortality or adolescent motherhood (eg, Social Institutions and Gender Index [11], and the Gender Inequality Index [8]), or women´s general well-being including safety from violence such as the Women, Peace and Security Index [12], have been proposed in the literature. However, most of these indices rely on national-level aggregate data and cannot be disaggregated by region or population subgroups, limiting within-country comparisons. Consideration of subgroups is very important to account for intersectionality, as it is known that socially marginalized women, including those who are poorer, rural, less educated or reside in fragile states, face greater risks to their health, well-being and even survival [13,14]. A few individual-level measures were also proposed [10,15,16], but most of these tools were specifically designed for a given country or a small region like East Africa (including only a few countries) [15], or a particular sector such as the Women's Empowerment in Agriculture Index, which has a particular focus on women who are working in agriculture [17].

In 2017, a survey-based women’s empowerment index (SWPER) was developed and validated using DHS data from 34 African countries [10], being the first individual-level indicator to enable comparisons between a large number of countries and over time. SWPER captures three empowerment domains indicative of assets and agency among partnered (either married or in a union) women:

1. Social independence: mainly composed by preconditions that enable women to achieve their goals (schooling attainment, access to information, age at pivotal life events and spousal asset differentials and access to information).
2. Decision-making: the extent of the woman’s participation is household decisions, which may also be considered a measure of instrumental agency.
3. Attitudes to violence: closely related to the concept of intrinsic agency, as a proxy for the woman’s incorporation of gender norms-related acceptability of violence.

The development of the SWPER used a similar conceptual framework further recently proposed by Miedema *et al* [15], which describes three domains of empowerment: enabling conditions, instrumental agency and intrinsic agency. Enabling conditions are considered preconditions that allow women to gain more power [18], and these correspond to the asset and opportunity constructs commonly focused upon in development economists’ definitions of empowerment [2,4]. Instrumental agency is the woman’s ability to make choices in the household, at family-level [15]. Intrinsic agency – or power within – is the process by which one develops a critical consciousness of one’s own aspirations, capabilities, and rights [15,19,20], and can be viewed as an asset and opportunity regarding safety of the environment or as a proxy for agency as safety would allow for greater ability to act on choice [15].

The SWPER uses individual-level data, allowing for assessing associations between empowerment and several health interventions and outcomes [10]. The SWPER also allows within-country and between-country comparisons, as well as time trend analyses. The items used to calculate the SWPER are available for over 60 countries with a DHS. The external validity and predictive value of the African SWPER has been demonstrated in terms of coverage of maternal and child interventions and use of modern contraception [10]. The SWPER attracted interest from the academic community and international agencies. In July 2018, a workshop with experts on women’s empowerment was held in Washington, DC, co-organized by PAHO and the Countdown to 2030 [21,22] to allow a comprehensive debate on definitions of women’s empowerment and the expansion of SWPER beyond Sub-Saharan Africa, especially in respect to the Latin American context (see **Box 1** for details). Feedback from the workshop prompted us to develop and test a global version of the SWPER that would allow its use in all LMICs.

Box 1 **Summary of the recommendations from the expert workshop held in Washington, DC, to discuss the adaptation of the SWPER for LMICs in Latin America and other world regions.**

In July 2018, an expert workshop was held in Washington, DC, bringing together a panel of experts with the aim of discussing how to best adapt the SWPER for use in Latin America and other world regions (https://equidade.org/news/123/expert-group-workshop-on-women-39-s-empowerment-in-the-lac-region). The workshop was co-organized by PAHO/WHO and the Countdown to 2030 and counted with the participation of over 20 experts from multilateral organizations and universities.

A series of suggestions for improving the indicator were made, notably:

1. Exclude the woman´s working status item, which was considered too simplistic to indicate whether or not work was empowering women, given that women may work because they are forced to work due to circumstance, and may not even be paid for this work;
2. For the questions on who decides on health care utilization and household expenses, give equal weight to joint decisions (with the partner or another person) and woman’s deciding alone;
3. Add items related to sexual and reproductive autonomy, decision-making on the use of the woman’s income, ownership of house or land; and access to technology, such as mobile phones; and
4. Include unpartnered women to allow the assessment of empowerment in this group.

The currently proposed version of the SWPER Global incorporates the first two recommendations. Given the constraints of the data currently available in surveys and the need for a readily available measure of women’s empowerment, the latter two recommendations were not incorporated at this time. We acknowledge these limitations and commit to continue our efforts to refine the indicator in the future.

## METHODS

Demographic and Health Surveys (DHS) are a reliable source of individual level information on socioeconomic characteristics, health, and development indicators in the context of LMICs. Since 1999, these surveys have been incorporating questions on women’s empowerment that potentially allows for comparisons within and between countries using an intersectional lens. We used data from the latest available DHS since 2000 for all available countries. For Mozambique, data from the 2011 survey was used because the 2015 survey lacked information on partner´s age and education. Six countries with a DHS were dropped because not all necessary items were available (Colombia, Turkey, Vietnam, Jordan, Yemen and Congo Brazzaville). Thus, 62 countries – 34 of which were already included in the SWPER for Africa [10] – were included in the analyses (**Table 1**), with a total sample of 662 835 partnered women.

**Table 1:** Composition patterns of the items that compose the SWPER domains with loadings’ equal or above 0.3*

				Domains																
				**Attitude to violence**					**Social independence**						**Decision-making**					
				**Items (key to item names below)†**																
**World region**	**Country**	**ISO code**	**Survey year**	**1**	**2**	**3**	**4**	**5**	**6**	**7**	**8**	**9**	**10**	**11**	**6**	**7**	**11**	**12**	**13**	**14**
**Pooled data set**				×	×	×	×	×	×	×	×	×						×	×	×
**South Asia**	Afghanistan	AFG	2015	×	×	×	×	×			×	×						×	×	×
	Bangladesh	BGD	2014	×	×	×	×	×	×	×	×	×						×	×	×
	India	IND	2015	×	×	×	×	×	×	×	×	×		×				×	×	×
	Maldives	MDV	2009	×	×	×	×	×		×	×	×						×	×	×
	Nepal	NPL	2016	×	×	×	×	×	×	×	×	×		×				×	×	×
	Pakistan	PAK	2012	×	×	×	×	×	×	×	×	×						×	×	×
**East Asia & Pacific**	Cambodia	KHM	2014	×	×	×	×	×			×	×						×	×	×
	Indonesia	IDN	2012	×	×	×	×	×	×	×	×	×						×	×	×
	Myanmar	MMR	2015	×	×	×	×	×		×	×	×						×	×	×
	Philippines	PHL	2017	×	×	×	×	×		×	×	×						×	×	×
	Timor-Leste	TLS	2016	×	×	×	×	×		×	×	×						×	×	×
**Europe & Central Asia**	Albania	ALB	2008	×	×	×	×	×		×	×	×						×	×	×
	Armenia	ARM	2015	×	×	×	×	×		×	×	×	×					×	×	×
	Azerbaijan	AZE	2006	×	×	×	×	×			×	×	×					×	×	×
	Kyrgyzstan	KGZ	2012	×	×	×	×	×		×	×	×		×				×	×	×
	Moldova	MDA	2005	×	×	×	×	×		×	×	×	×					×	×	×
	Tajikistan	TJK	2012	×	×	×	×	×			×	×				×	×	×	×	×
	Ukraine	UKR	2007	×	×	×	×	×		×	×	×						×	×	×
**Middle East & North Africa**	Egypt	EGY	2014	×	×	×	×	×		×	×	×						×	×	×
	Morocco	MAR	2003	×	×	×	×	×		×	×	×						×	×	×
**West & Central Africa**	Benin	BEN	2011	×	×	×	×	×	×	×	×	×						×	×	×
	Burkina Faso	BFA	2010	×	×	×	×	×	×	×	×	×						×	×	×
	Cameroon	CMR	2011	×	×	×	×	×	×	×	×	×						×	×	×
	Chad	TCD	2014	×	×	×	×	×	×	×	×	×						×	×	×
	Congo DR	COD	2013	×	×	×	×	×	×	×	×	×		×				×	×	×
	Cote d’Ivoire	CIV	2011	×	×	×	×	×	×	×	×	×						×	×	×
	Gabon	GAB	2012	×	×	×	×	×	×	×	×	×		×				×	×	×
	Gambia	GMB	2013	×	×	×	×	×	×	×	×	×						×	×	×
	Ghana	GHA	2014	×	×	×	×	×	×	×	×	×						×	×	×
	Guinea	GIN	2012	×	×	×	×	×	×	×	×	×						×	×	×
	Liberia	LBR	2013	×	×	×	×	×	×	×	×	×		×				×	×	×
	Mali	MLI	2012	×	×	×	×	×	×	×	×	×						×	×	×
	Niger	NER	2012	×	×	×	×	×	×	×	×	×						×	×	×
	Nigeria	NGA	2013	×	×	×	×	×	×	×	×	×						×	×	×
	Sao Tome & Principe	STP	2008	×	×	×	×	×	×	×	×	×		×				×	×	×
	Senegal	SEN	2017	×	×	×	×	×	×	×	×	×						×	×	×
	Sierra Leone	SLE	2013	×	×	×	×	×	×	×	×	×						×	×	×
	Togo	TGO	2013	×	×	×	×	×	×	×	×	×						×	×	×
**Eastern & Southern Africa**	Angola	AGO	2015	×	×	×	×	×	×	×	×	×		×				×	×	×
	Burundi	BDI	2016	×	×	×	×	×		×	×	×						×	×	×
	Comoros	COM	2012	×	×	×	×	×	×	×	×	×						×	×	×
	Ethiopia	ETH	2016	×	×	×	×	×	×	×	×	×						×	×	×
	Kenya	KEN	2014	×	×	×	×	×	×	×	×	×						×	×	×
	Lesotho	LBN	2014	×	×	×	×	×	×	×	×	×						×	×	×
	Madagascar	MDG	2008	×	×	×	×	×	×	×	×	×						×	×	×
	Malawi	MWI	2015	×	×	×	×	×	×	×	×	×						×	×	×
	Mozambique	MOZ	2011	×	×	×	×	×	×	×	×	×						×	×	×
	Namibia	NAM	2013	×	×	×	×	×	×	×	×	×		×				×	×	×
	Rwanda	RWA	2014	×	×	×	×	×		×	×	×						×	×	×
	Eswatini	SWZ	2006	×	×	×	×	×	×	×	×	×						×	×	×
	Tanzania	TZA	2015	×	×	×	×	×	×	×	×	×		×				×	×	×
	Uganda	UGA	2016	×	×	×	×	×	×	×	×	×		×				×	×	×
	Zambia	ZMB	2013	×	×	×	×	×	×	×	×	×						×	×	×
	Zimbabwe	ZWE	2015	×	×	×	×	×	×	×	×	×						×	×	×
**Latin America & Caribbean**	Bolivia	BOL	2008	×	×	×	×	×		×	×	×						×	×	×
	Dominican Republic	DOM	2013	×	×	×	×	×		×	×	×						×	×	×
	Guatemala	GTM	2014	×	×	×	×	×		×	×	×			×	×		×	×	×
	Guyana	GUY	2009	×	×	×	×	×	×	×	×	×		×				×	×	×
	Haiti	HTI	2016	×	×	×	×	×	×	×	×	×						×	×	×
	Honduras	HND	2011	×	×	×	×	×			×	×				×		×	×	×
	Nicaragua	NIC	2001	×	×	×	×	×		×	×	×						×	×	×
	Peru	PER	2016	×	×	×	×	×		×	×	×				×		×	×	×

*Some items are repeated in the table because they presented a loading equal or above 0.3 in different domains across the countries.

†Key to items numbers: Beating justified if: (1) wife goes out without telling husband; (2) wife neglects the children; (3) wife argues with husband; (4) wife refuses to have sex with husband; (5) wife burns the food. Item (6) frequency of reading newspaper or magazine; (7) woman’s education; (8) age at 1st birth; (9) age at 1st cohabitation; (10) age difference: woman's minus husband's age; (11) education difference: woman’s minus husband’s years of schooling; who usually decides on: (12) respondent’s health care; (13) large household purchases; (14) visits to family or relatives.

### Content validity

The SWPER Global was developed using similar methods to what was done for the SWPER for Africa. In that case, we used 15 items available in DHS surveys (see **Table 2**): five items related to the woman’s opinion on whether a husband beating his wife is justified in specific situations (wife goes out without telling husband; wife neglects the children; wife argues with the husband; wife refuses to have sex with the husband; and if she burns the food), frequency of reading newspaper or magazine, her education in completed years of schooling, age at first birth and at first cohabitation, age and education difference between the woman and her husband, three questions on the woman’s participation in decisions about seeking health care for herself, large household purchases and visits to family and relatives, and whether the woman worked in the last year. Except for the items related to age and education that are continuous, all other items are ordinal with higher scores given to categories considered of higher empowerment level (the codes are provided in **Table 2**). The scores were derived through principal components analyses (PCA) using surveys from 34 African countries [10]. We obtained an indicator with three domains: (1) attitude to violence, based on the five questions asking the women’s opinion on whether a husband beating the wife is justified in specific situations; (2) social independence, comprising access to information, educational attainment, age at marriage and first child, and differences in age and education to the cohabiting partner; and (3) decision-making, based on the questions related to who makes decisions in the household and to the women’s work.

**Table 2:** Items used in each domain of the African-oriented survey-based women’s empowerment (SWPER) index and the changes made in the global version of the index, according to gender experts’ recommendations

Item (v)	Code or unit	Changes
**Attitude to violence domain**		
1. Beating justified if wife goes out without telling husband	Yes = -1; Don’t know = 0; No = 1	No changes
2. Beating justified if wife neglects the children	Yes = -1; Don’t know = 0; No = 1	No changes
3. Beating justified if wife argues with husband	Yes = -1; Don’t know = 0; No = 1	No changes
4. Beating justified if wife refuses to have sex with husband	Yes = -1; Don’t know = 0; No = 1	No changes
5. Beating justified if wife burns the food	Yes = -1; Don’t know = 0; No = 1	No changes
**Social independence domain:**		
6. Frequency of reading newspaper or magazine	Not at all = 0;	No changes
	<once a week = 1;	
	≥once a week = 2	
7. Woman education in completed years of schooling	Years	No changes
8. Age of woman at first birth*	Years	No changes
9. Age at first cohabitation	Years	No changes
10. Age difference: woman’s minus husband’s age	Years	No changes
11. Education difference: woman’s minus husband’s years of schooling	Years	No changes
**Decision-making domain:**		
12. Who usually decides on respondent's health care	Husband or other alone = -1; joint decision = 0; respondent alone = 1	**Husband or other alone = -1; Joint decision or respondent alone = 1**
13. Who usually decides on large household purchases	Husband or other alone = -1; joint decision = 0; respondent alone = 1	**Husband or other alone = -1; Joint decision or respondent alone = 1**
14. Who usually decides on visits to family or relatives	Husband or other alone = -1; joint decision = 0; respondent alone = 1	**Husband or other alone = -1; Joint decision or respondent alone = 1**
X. Respondent worked in last 12 mo	No = 0; In the past year = 1; Have a job, but on leave last 7 d = 2; Currently working = 2	**Item excluded**

*This item age at first birth was imputed for those women who had not had a child, please see section 1.1 in the **Online Supplementary Document** for details.

Considering the expert workshop recommendations (Panel 1), we excluded the item that indicates whether the woman worked in the last year and changed the categorization of the decision-making related variables, so that equal weights were given for joint decisions (with the partner or another person) and woman’s sole decision (**Table 2**).

### Construct validity

Following a similar methodology used for Africa [10], we used PCA to identify the empowerment domains and estimate the items loadings for each of the 62 countries separately, after applying varimax rotation. We then applied the same strategy to a pooled data set combining all countries, to derive a single indicator. Loadings of 0.3 or more are generally considered good [23]. The patterns emerged were compared in terms of items with loadings of 0.3 or higher in each empowerment domain across countries to check for consistency, and Pearson correlation coefficients between the scores derived for each individual country and global scores derived from the combined data for each empowerment domain of the indicator were estimated. By doing so, we aimed to evaluate whether these two approaches would present consistent estimates of individual empowerment levels.

### External validity

To assess the external validity of the SWPER for this extended set of LMICs, Spearman correlation coefficients were calculated between the resulting pooled score and two widely used indices: the Gender Development Index (GDI) and the Gender Inequality Index (GII) [8]. In this case, Spearman correlation was used because we were interested in the correlation between the ranking of the countries using these different measures, rather than the correlation between the scores.

The equations to calculate the SWPER Global are provided in the online supplementary document with a step-by-step explanation. To allow researchers to choose the reference population and standardize the SWPER scores according to the world region they consider more suitable for a given study, we also calculated the means and standard deviations of the SWPER Global domains for each world region additionally to the scores estimated for all countries together (Table S2 in the **Online Supplementary Document**). With that, a researcher interested in India, for example, could choose South Asia or the SWPER Global (all countries together) as reference population to standardize the SWPER scores. In the Results section below, we used the global mean for LMICs and standard deviation to standardize the scores. Based on the distribution of scores, we also proposed a categorization of the SWPER domains in three groups: low, medium and high empowerment level. For the social independence domain, for which the distribution to a normal curve, the scores were divided into terciles. In contrast, the attitude to violence and decision-making domains present multimodal distributions, which were considered to define the cut-offs (see Figure S1 and Table S3 in the **Online Supplementary Document**).

All analyses were performed using the statistical software Stata (StataCorp. 2017. Stata Statistical Software: Release 15.1. College Station, TX: StataCorp LLC). The calculation of average scores at country level took into account the surveys’ sample design. DHS are public sources of information and ethical approval has already been obtained from each country by the time of the survey conduction.

## RESULTS

The same three domains identified in the SWPER for Africa were observed in the global analyses: attitude to violence, social independence and decision making. The cross-country consistency among items composing each domain of the SWPER Global can be assessed through **Table 1**, where we present items with a loading of 0.3 or more (henceforth referred to as high loadings). All countries presented the exact same pattern of items identified in the attitude to violence domain, which included all the questions related to the woman’s opinion on whether a husband is justified in beating the wife in five different circumstances. Items composing the decision-making domain was also consistent across countries with higher loadings for the three questions related to the women´s involvement in household decisions, with only four countries presenting high-loading items that were not related to participation in household decisions. Three of them were from Latin America (Honduras and Peru, that also included woman’s education; and Guatemala, that included education and frequency of reading newspaper or magazine) and one from Europe & Central Asia (Tajikistan, that also included woman’s education and education difference between the woman and her husband). For the social independence domain, a greater variability in the patterns of items identified was observed. Countries from Africa and South Asia were the most stable in terms of the patterns for this domain (composed by the frequency of reading newspaper/magazine, women’s education, age the first birth, age at first cohabitation, and age and education differentials in relation to the partner). In the other four regions of the world, most countries presented low loadings for the frequency of reading newspapers or magazines. Education showed low loadings in only five countries (Azerbaijan, Tajikistan, Afghanistan, Cambodia and Honduras). The items related to pivotal events (age at first birth and at first cohabitation) presented high loadings in all countries. Age differentials to the partner had high loadings in three Europe and Central Asia countries (Armenia, Azerbaijan and Moldova) and education differentials to the partner in 12 out of the 62 countries, but without a clear pattern.

In **Table 3** we present the correlations between the three SWPER domain scores calculated separately using principal component analyses in each country, and the global scores calculated using the pooled data. Cells are colored from yellow to dark green cells showing the strength of the correlations. Even though the item patterns presented in **Table 1** were not entirely consistent for some countries (ie, all items presenting loading ≥0.3 in the same empowerment domain), the correlations between the country-specific and the SWPER Global scores were very high, equaling 0.89 or more. All countries presented correlations greater than 0.91 for all three SWPER domains, except for Gabon and Liberia where the correlations for social independence were 0.89 and 0.90, respectively.

**Table 3:** Pearson correlation between the country-specific women’s empowerment measure and the SWPER global index for each domain

			Pearson correlation (r)		
**World region**	**Country**	**Year**	**Attitude to violence**	**Social independence**	**Decision Making**
**South Asia**	Afghanistan	2015	0.9923*	0.9545‡	0.9820‡
	Bangladesh	2014	0.9968*	0.9898‡	0.9936*
	India	2015	0.9983*	0.9935*	0.9940*
	Maldives	2009	0.9976*	0.9819‡	0.9687‡
	Nepal	2016	0.9872‡	0.9896‡	0.9911*
	Pakistan	2012	0.9996*	0.9966*	0.9950*
**East Asia & Pacific**	Cambodia	2014	0.9901*	0.9776‡	0.9685‡
	Indonesia	2012	0.9918*	0.9930*	0.9928*
	Myanmar	2015	0.9864‡	0.9930*	0.9636‡
	Philippines	2017	0.9753‡	0.9911*	0.9862‡
	Timor-Leste	2016	0.9942*	0.9933*	0.9813‡
**Europe & Central Asia**	Albania	2008	0.9959*	0.9807‡	0.9743‡
	Armenia	2015	0.9846‡	0.9821‡	0.9848‡
	Azerbaijan	2006	0.9884‡	0.9752‡	0.9960*
	Kyrgyzstan	2012	0.9971*	0.9620‡	0.9920*
	Moldova	2005	0.9908*	0.9726‡	0.9579‡
	Tajikistan	2012	0.9994*	0.9807‡	0.9974*
	Ukraine	2007	0.9670‡	0.9890‡	0.9737‡
**Middle East & North Africa**	Egypt	2014	0.9978*	0.9943*	0.9955*
	Morocco	2003	0.9900*	0.9981*	0.9955*
**West & Central Africa**	Benin	2011	0.9977*	0.9569‡	0.9968*
	Burkina Faso	2010	0.9991*	0.9784‡	0.9758‡
	Cameroon	2011	0.9992*	0.9938*	0.9965*
	Chad	2014	0.9934*	0.9677‡	0.9778‡
	Congo DR	2013	0.9964*	0.9898‡	0.9972*
	Cote d’Ivoire	2011	0.9989*	0.9526‡	0.9956*
	Gabon	2012	0.9973*	0.8895§	0.9901*
	Gambia	2013	0.9986*	0.9858‡	0.9857‡
	Ghana	2014	0.9986*	0.9906*	0.9979*
	Guinea	2012	0.9887‡	0.9639‡	0.9950*
	Liberia	2013	0.9990*	0.8959§	0.9944*
	Mali	2012	0.9966*	0.9463†	0.9943*
	Niger	2012	0.9991*	0.9479†	0.9944*
	Nigeria	2013	0.9988*	0.9938*	0.9971*
	São Tome & Principe	2008	0.9955*	0.9649‡	0.9955*
	Senegal	2017	0.9988*	0.9772‡	0.9821‡
	Sierra Leone	2013	0.9985*	0.9246†	0.9956*
	Togo	2013	0.9983*	0.9840‡	0.9913*
**Eastern & Southern Africa**	Angola	2015	0.9981*	0.9264†	0.9786‡
	Burundi	2016	0.9996*	0.9920*	0.9936*
	Comoros	2012	0.9949*	0.9876‡	0.9844‡
	Eswatini	2006	0.9890‡	0.9684‡	0.9870‡
	Ethiopia	2016	0.9991*	0.9896‡	0.9976*
	Kenya	2014	0.9989*	0.9962*	0.9948*
	Lesotho	2014	0.9976*	0.9945*	0.9837‡
	Madagascar	2008	0.9928*	0.9906*	0.9734‡
	Malawi	2015	0.9962*	0.9944*	0.9970*
	Mozambique	2011	0.9954*	0.9895‡	0.9833‡
	Namibia	2013	0.9966*	0.9202†	0.9932*
	Rwanda	2014	0.9990*	0.9992*	0.9883‡
	Tanzania	2015	0.9979*	0.9754‡	0.9969*
	Uganda	2016	0.9995*	0.9822‡	0.9835‡
	Zambia	2013	0.9992*	0.9899‡	0.9903*
	Zimbabwe	2015	0.9990*	0.9893‡	0.9918*
**Latin America & Caribbean**	Bolivia	2008	0.9897‡	0.9938*	0.9683‡
	Dominican Republic	2013	0.9414†	0.9946*	0.9883‡
	Guatemala	2014	0.9877‡	0.9675‡	0.9190†
	Guyana	2009	0.9967*	0.9702‡	0.9927*
	Haiti	2016	0.9929*	0.9853‡	0.9683‡
	Honduras	2011	0.9927*	0.9711‡	0.9242†
	Nicaragua	2001	0.9939*	0.9950*	0.9897‡
	Peru	2016	0.9643‡	0.9904*	0.9372†

Note: Cells are coded according to the correlations: §(r <0.900); †(r = 0.900 to <0.950); ‡(r = 0.950 to <0.990), *(r ≥0.990).

**Figure 1:**
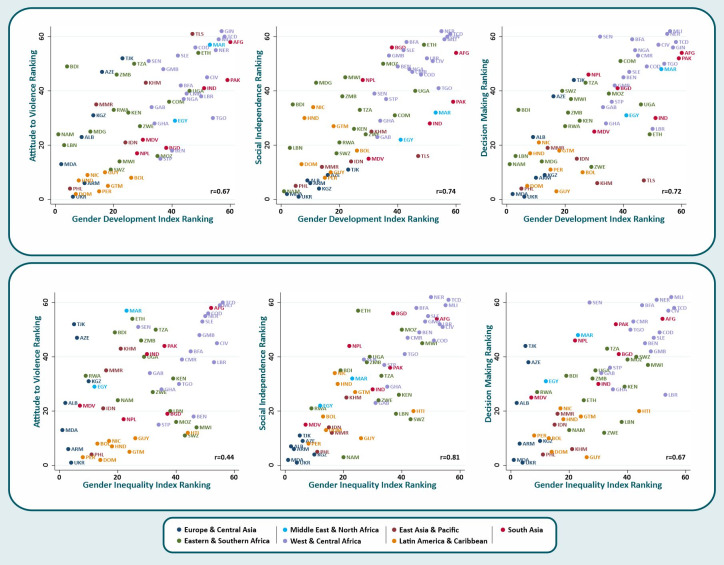
Scatter plot showing the SWPER domains ranking against the Gender Development Index and the Gender Inequality Index rankings with the Spearman correlation (r) indicated in the bottom right of each plot.

**Figure 1** shows scatter plots of the country ranks for the three SWPER domains against the country ranks for the GDI and the GII, providing evidence on the external validity of the SWPER. The correlation coefficients with the GDI (upper graphs) were 0.74, 0.72 and 0.67 for social independence, decision-making and attitude to violence, respectively. In the lower graphs, the SWPER rankings are plotted against the GII rankings. In this case, the Spearman correlations were 0.81, 0.67 and 0.44, respectively. **Figure 1** shows that Latin America & Caribbean and Europe & Central Asia tend to present higher ranks in all empowerment domains while West & Central Africa, Eastern & Southern Africa and some countries from South Asia presented the lowest rankings.

Generally, Europe & Central Asia and Latin America & Caribbean show the higher mean scores in the three empowerment domains (**Table 4**). The African regions and South Asia presented the lower mean scores, with West & Central Africa showing the lowest average scores in all three empowerment domains. East Asia and Pacific presented high mean scores for social independence and decision making but scored below the average for attitude to violence.

**Table 4:** Mean and standard error of the SWPER global scores in each domain according to the world region*

Region	Number of countries	Attitude to violence		Social independence		Decision-making	
**Mean**	**Std. error**	**Mean**	**Std. error**	**Mean**	**Std. error**
South Asia	6	0.007	0.184	-0.155	0.149	0.072	0.139
East Asia & Pacific	5	-0.016	0.266	0.440	0.106	0.774	0.063
Europe & Central Asia	7	0.209	0.181	0.809	0.064	0.672	0.164
Middle East & North Africa	2	-0.281	0.455	0.140	0.149	0.161	0.247
West & Central Africa	18	-0.288	0.122	-0.403	0.071	-0.260	0.111
Eastern & Southern Africa	16	0.078	0.087	-0.006	0.088	0.388	0.070
Latin America and Caribbean	8	0.589	0.035	0.322	0.098	0.722	0.051

*These analyses used the countries as units of analyses, so they are not affected by the country population size.

## DISCUSSION

We explored whether a slightly modified version of the original SWPER, previously validated for African countries [10], could be used as an indicator of women´s empowerment across LMICs. 14 items identified from the previous version, that allow the assessment of empowerment in three dimensions (attitude to violence, social independence, and decision-making), were pre-selected for the SWPER Global validation process. The PCA analysis, showed consistency across countries in terms of patterns of components retained and item loadings for the attitude to violence and decision-making domains. For the social independence domain, we found more variability in terms of the item loadings. In particular, women’s education had low loadings in regions with higher educational levels, and therefore less variability in educational levels. Notwithstanding, the correlation between the country-specific and the SWPER Global scores were above 0.89 for all three empowerment domains in all 62 countries included in the analysis.

Two important changes from the previous version of the SWPER were made based on the workshop held with experts on women’s empowerment. The first was to remove the women’s working status as it was agreed that there are many reasons why women work, which are not necessarily related to their own choices. Also, work may not be empowering, depending on the working conditions, and on whether or not women are formally employed, are entitled to maternity leave, are paid for their work, and are able to decide what to do with their earnings. Our preliminary analyses (results not shown) showed that in several countries the work item had negative loadings in the PCA, suggesting that working could be indicative of lower empowerment levels. This inconsistent behavior has also been demonstrated in the literature. For example, women who were working and paid in cash in Egypt were at lower risk of experiencing intimate partner violence, while in India, Peru and Iran their risk was higher [24,25]. The second change in the SWPER Global was to give equal weights to women´s involvement in household decisions, whether they were taken only by the women or jointly with her the partner. In the original version of the SWPER, a decision made by the woman alone was given a higher score than a joint decision with the partner. However, there was consensus among experts that joint decisions on household issues such as purchases and visits to family or friends were thought to be a potential reflection of gender equality [26]. These content modifications were essential to refine and expand the index to a global context including all LMICs.

Our findings show the SWPER Global is a suitable common measure of women’s empowerment for LMICs, addressing the need for a single consistent survey-based indicator of women´s empowerment that allows to track progress over time and across countries at the individual and country levels. The external validity assessment showed overall strong correlations between country rankings in the SWPER Global with their rankings in the GDI and the GII which capture distinct aspects of gender inequalities at the country level. Construct validity of the SWPER was also previously assessed at the individual level, through its positive association with modern contraceptive use, institutional delivery, stunting [10] and, more recently, with child developmental outcomes [27] and neonatal, infant and under-5 mortality [28]. In the current manuscript, we chose not to present associations between the SWPER Global and health outcomes as other recently published studies have already shown its positive associations with the coverage of reproductive, maternal, newborn and child health interventions [29]; and negative association with experience of psychological and physical or sexual violence. [30]These results reassure the internal validity of the SWPER Global and its potential to widen the research on the effects of women’s empowerment on health interventions and outcomes.

After the first version of the SWPER was published in 2017, other similar measures of women´s empowerment in the context of Africa have been proposed in the literature. In 2018, Asaolu et al. [31], using DHS from nineteen countries across Sub-Saharan Africa, identified attitude towards violence, labor force participation, education, and access to health care as valid domains of empowerment although some variations between sub-regions were observed (eg, in East Africa education was surprisingly not a relevant component of women´s empowerment) [31]. Also in 2018, Miedema et al. [15] tested the cross-national invariance of women´s empowerment using DHS data from five countries in East Africa and identified three domains of women´s empowerment capturing women´s human/social assets, attitudes related to wife abuse, and women´s participation decision applicable across countries, very similar to what was presented in the original SWPER paper [10]. These other efforts highlight the importance of the topic and how different approaches can lead to different but similar results. Also, how diverse priorities also lead to different approaches. In our case, we compromised on country specificity to obtain an indicator that allows for comparative analysis and monitoring across a large array of countries.

The development of a global common measure of women´s empowerment, however, represents a huge challenge, given its complex and multidimensional constructs as well as their variability across different societies with contextually specific gender vulnerabilities. As a result, the SWPER Global has limitations, some already previously recognized and discussed in our previous publication [32]. Empowerment is not primarily an outcome, but a process that includes critical consciousness, aspiration, voice, choice, and change [33,34]; and as such, there are elements enabling or limiting it that have not been considered in the SWPER [32]. Decision-making assesses control but not choice, nor risk for sanctions or backlash based on choices made. Inclusion of aspects such as personal ownership of assets, economic participation, and opportunities and participation in governance processes were also pointed in the expert workshop as important measures of empowerment that are not captured by the SWPER. Some of these items are not available in DHS surveys and would require different data sources. However, there is a growing number of measures related to assets, opportunity and agency that are being added to DHS with potential to be incorporated in future updates of the SWPER Global. These include mobile phone ownership, internet access, bank accounts, and decision-making on how to spend personal earnings. As these are only available for a few recent surveys, their inclusion in the SWPER at this point would markedly reduce the number of countries for which the index could be calculated. Also, more items are needed to capture additional domains of women’s empowerment, especially in contexts with higher socioeconomic development, including (but not being restricted to) economic empowerment, sexual and reproductive empowerment, power relations outside marriage including political engagement or influence, social and occupational leadership and positioning, and freedom of movement and safety at the individual level.

Another important limitation is that the index is limited to women married or in a union and therefore leaves out sizeable groups of women in some countries (Table S4 in the **Online Supplementary Document**). African and Asian countries tend to have higher proportions of married adolescent girls, which may affect comparisons of empowerment levels across world regions and require age-stratified analyses. The development and validation of a measure that includes both partnered and unpartnered women, however, would require a very different approach, which is beyond the scope of the proposed indicator based on national health surveys for which most of the relevant information are only available for women that are married or in a union. Lastly, it is important to note that due to data availability, the countries included in our analysis represent 48% of all LMICs, ranging from over 70% of the countries from South Asia, West and Central Africa and Eastern and Southern Africa to 14% of the countries from Middle East and North Africa (Table S5 in the **Online Supplementary Document**). Given the little difference found in the behavior of the SWPER between the original version for Africa and the current, including all available LMICs, it is unlikely that the inclusion of more countries would lead to relevant changes to the indicator we proposed.

## CONCLUSIONS

Acknowledging the current limitations of the SWPER Global, particularly in light of the recommendations from the expert workshop, efforts must continue to improve the indicator. We commend the DHS efforts to include questions related to women’s empowerment in the surveys, but having more information will be fundamental to advance in this direction either capturing other domains of women’s empowerment or improving the ones already measured with the SWPER. Several measures related to assets, opportunity and agency are already being added to the most recent DHS and will potentially be incorporated in future updates of the SWPER Global. However, more items related to domains like economic empowerment, sexual and reproductive empowerment, power relations outside marriage, leadership and positioning, and freedom of movement should be considered for inclusion in the surveys to provide a more robust measure of women’s empowerment, consistent with the SDG5 agenda. Another future challenge will be to develop another indicator that includes unpartnered women and enables comparison with the partnered ones, as well as the evaluation of inequalities between subgroups of women. Nevertheless, the SWPER Global is a pioneer indicator of women´s empowerment based on individual-level survey data from LMICs, which enables comparability between countries from all world regions and over time. It represents an advance over other global gender and development indices by including a domain on attitudes towards violence against women, a prevalent form of human rights violation as well as an important universally recognized aspect of gender inequality worldwide. Use of the SWPER Global will contribute to monitoring the progress towards SDG5, in terms of gender equality and empowerment at the national and sub-national levels. It also allows quantification of the role of empowerment in terms of health, nutrition and developmental outcomes of women and children worldwide.

## Additional material

Online Supplementary Document

## References

[1] Van Eerdewijk A, Newton J, Tyszler M, Wong F, Vaast C. White Paper: A Conceptual Model of Women and Girls’ Empowerment. Amsterdam: 2016.

[2] Kabeer N Gender equality and women’s empowerment: A critical analysis of the third Millennium Development Goal. Gend Dev. 2005;13:13-24. 10.1080/13552070512331332273

[3] United Nations Development Programme. UNDP gender equality strategy 2014-2017. New York: 2017.

[4] Alsop R, Bertelsen M, Holland J. Empowerment in Practice. Washington, DC: The World Bank; 2005.

[5] Zimmerman MA Psychological empowerment: Issues and illustrations. Am J Community Psychol. 1995;23:581-99. 10.1007/BF02506983 8851341

[6] Bandura A Human agency in social cognitive theory. Am Psychol. 1989;44:1175. 10.1037/0003-066X.44.9.1175 2782727

[7] World Economic Forum. The Global Gender Gap Report. Geneva: WEF; 2017.

[8] United Nations Development Programme. Human Development Report 2016, Human Development for Everyone. New York: 2016.

[9] United Nations Development Programme. Human Development Indices and Indicators - 2018 Statistical Update. Vol. 27. New York: 2018. http://hdr.undp.org/sites/default/files/2018_human_development_statistical_update.pdf

[10] Ewerling F Lynch JW Victora CG van Eerdewijk A Tyszler M Barros AJD The SWPER index for women’s empowerment in Africa: development and validation of an index based on survey data. Lancet Glob Health. 2017;5:e916-23. 10.1016/S2214-109X(17)30292-9 28755895 PMC5554795

[11] Organisation for Economic Co-operation and Development (OECD). Social Institutions Gender Index: Methodological Background Paper. Paris: 2014.

[12] GIWPS. (Georgetown Institute for Women, Peace and Security) and PRIO (Peace Research Institute Oslo). Women, peace and security index 2017/18: Tracking sustainable peace through inclusion, justice, and security for women. 2017.

[13] Hay K McDougal L Percival V Henry S Klugman J Wurie H Disrupting gender norms in health systems: making the case for change. Lancet. 2019;393:2535-49. 10.1016/S0140-6736(19)30648-8 31155270 PMC7233290

[14] Heise L Greene ME Opper N Stavropoulou M Harper C Nascimento M Gender inequality and restrictive gender norms: framing the challenges to health. Lancet. 2019.393:2440-54. 10.1016/S0140-6736(19)30652-X 31155275

[15] Miedema SS Haardörfer R Girard AW Yount KM Women’s empowerment in East Africa: Development of a cross-country comparable measure. World Dev. 2018;110:453-64. 10.1016/j.worlddev.2018.05.031

[16] Upadhyay UD Karasek D Women’s empowerment and ideal family size: An examination of DHS empowerment measures in Sub-Saharan Africa. Int Perspect Sex Reprod Health. 2012;38:78-89. 10.1363/3807812 22832148

[17] Alkire S Meinzen-Dick R Peterman A Quisumbing A Seymour G Vaz A The women’s empowerment in agriculture index. World Dev. 2013;52:71-91. 10.1016/j.worlddev.2013.06.007

[18] Malhotra A, Schuler SR, Boender C. Measuring women’s empowerment as a variable in international development. Backgr. Pap. Prep. World Bank Work. Poverty Gend. New Perspect. 2002:28.

[19] Batliwala S. The meaning of women’s empowerment: New concepts from action. In: Sen G, Germain A, Chen LC, editors. Popul. policies reconsidered Heal. Empower. rights, vol. 17, Boston: Harvard University, Harvard Center for Population and Development Studies.; 1994.

[20] Kabeer N Resources, agency, achievements: Reflections on the measurement of women’s empowerment. Dev Change. 1999;30:435-64. 10.1111/1467-7660.00125

[21] Pan American Health Organization. Pan American Health Organization (PAHO) website n.d. https://www.paho.org/ (accessed July 10, 2020).

[22] Countdown to 2030. Countdown to 2030 website n.d. http://countdown2030.org/ (accessed July 10, 2020).

[23] Hair JF, Black WC, Babin BJ, Anderson RE, Tatham RL. Multivariate data analysis. vol. 5. Upper Saddle River, NJ: Prentice Hall.; 1998.

[24] Vyas S Watts C How does economic empowerment affect women’s risk of intimate partner violence in low and middle income countries? A systematic review of published evidence. J Int Dev. 2009;21:577-602. 10.1002/jid.1500

[25] Paul S Women’s labour force participation and domestic violence: Evidence from India. J South Asian Dev. 2016;11:224-50. 10.1177/0973174116649148

[26] Osamor PE Grady C Autonomy and couples’ joint decision-making in healthcare. BMC Med Ethics. 2018;19:3. 10.1186/s12910-017-0241-6 29325536 PMC5765707

[27] Ewerling F Lynch J Mittinty M Raj A Victora CG Coll CVN The Impact of Women’s Empowerment on Their Children’s Early Development in 26 African Countries. J Glob Health. 2020;10:020406 10.7189/jogh.10.020406 33214898 PMC7649042

[28] Doku DT Bhutta ZA Neupane S Associations of women’s empowerment with neonatal, infant and under-5 mortality in low-and /middle-income countries: meta-analysis of individual participant data from 59 countries. BMJ Glob Health. 2020;5:e001558. 10.1136/bmjgh-2019-001558 32133162 PMC7042599

[29] World Health Organization. Primary health care on the road to universal health coverage: 2019 monitoring report. Geneva: WHO; 2019.

[30] Coll CVN, Ewerling F, García-Moreno C, Hellwig F, Barros AJD. Intimate partner violence in 46 low-income and middle-income countries: An appraisal of the most vulnerable groups of women using national health surveys. BMJ Glob Heal. 2020;5. 10.1136/bmjgh-2019-002208 PMC7042580 32133178

[31] Asaolu IO Alaofè H Gunn JKL Adu AK Monroy AJ Ehiri JE Measuring women’s empowerment in Sub-Saharan Africa: Exploratory and Confirmatory Factor Analyses of the demographic and health surveys. Front Psychol. 2018;9:994. 10.3389/fpsyg.2018.00994 29971030 PMC6018402

[32] Raj A Gender Empowerment Index: a choice of progress or perfection. Lancet Glob Health. 2017;5:e849-50. 10.1016/S2214-109X(17)30300-5 28755893

[33] Kabeer N Between affiliation and autonomy: navigating pathways of women’s empowerment and gender justice in rural Bangladesh. Dev Change. 2011;42:499-528. 10.1111/j.1467-7660.2011.01703.x 21898946

[34] Klugman J, Tyson L. Leave no one behind: a call to action for gender equality and women’s economic empowerment. Report of the UN Secretary General: 2016.

