# Supplementary material for: SWPER Global: A survey-based women's empowerment index expanded from Africa to all low- and middle-income countries
Source: J Glob Health. 2020 Nov 8;10(2):020434. doi: 10.7189/jogh.10.020434 (PMC7699005; doi:10.7189/jogh.10.020434)
Supplement: Online Supplementary Document [file jogh-10-020434-s001.pdf]

A survey-based women's empowerment index to be used in all low- and middle-income country surveys: the SWPER goes global

Online Supplementary Document

How to calculate the SWPER for a specific survey

The equation used to estimate individual standardized scores for each of the principal component analysis (PCA) j components is given by:

$$S_{ij} = \frac{\left[[\lambda_{1j}(x_{1i} - \bar{x}_1)] + [(\lambda_{2j}(x_{2i} - \bar{x}_2)] + \cdots + [\lambda_{14j}(x_{14i} - \bar{x}_{14})]\right]}{\sigma_j} \quad (1)$$

where S_{ij} are the individual standardized scores for individual i and component j; $x_{1j}, \ldots, x_{14j}$ are the individual values for items x1-x14 included in the PCA analyses; σ_j are the standard deviations of the predicted scores of each component j. The weight given to each of the 15 items in each component j is defined as:

$$\lambda_{vj} = \frac{\varphi_{vj}}{\sigma_v} \quad (2)$$

Where φ_{vj} is the PCA loading for each of the items v in each domain j and σ_v is the standard deviation of each item v in the combined dataset.
By using simple algebra, we can simplify the equation above to:

$$S_{ij} = \frac{\left[-(\sum_{v=1}^{14} \lambda_{vj}\,\bar{x}_v) + \sum_{v=1}^{14}(\lambda_{vj}x_{vi})\right]}{\sigma_j} \quad (3)$$

Please, follow the next steps to calculate the standardized individual SWPER scores for any low- and middle-country (LMIC) of your interest[1]:

1. Recode items

The first step is to recode the items as it is shown in Table 2, presented in the article.

1.1. Imputation of woman’s age at first birth

We used single hotdeck imputation to impute the age at first birth for nulliparous women, clustering women according to their age at first cohabitation. In many countries the number of women that had the first cohabitation later in life was very small, so we generated a new variable of age at first cohabitation to use in the imputation where the maximum age was set at 33+ years.

2. Calculate the individual scores

Using the equations below, it is possible to estimate the scores for the three SWPER domains:

$$Score_{\,Attitude\ to\ violence_i} = (-1.202) + \sum_{v=1}^{14}(\lambda_{v1}x_{vi})$$

$$Score_{\,Social\ independence_i} = (-5.661) + \sum_{v=1}^{14}(\lambda_{v2}x_{vi})$$

$$Score_{\,Decision-making_i} = (-0.168) + \sum_{v=1}^{14}(\lambda_{v3}x_{vi})$$

Where x_{vi} is the value of items v for each individual i and $\lambda_{v1} - \lambda_{v3}$ are the item weights, that can be found in Table S1.

[1] A Stata do-file with all procedures required for the calculation of the SWPER Index scores is available from the Dropbox link <https://www.dropbox.com/sh/cw6e2th24l4ausa/AAC792VQx3CL7CuIdLwTxKiWa?dl=0>.

3. Standardize the calculated SWPER scores

The last step in the calculation of the individual scores is their standardization. You can decide whether to (1) use the SWPER global mean and standard deviations to standardize the scores (zero score represents the average LMICs mean); or (2) use a specific region mean and standard deviation, then having an empowerment measure that is standardized for that specific region (zero value represents the average empowerment level of that specific region). To standardize the SWPER score the procedure is simple. One should just subtract the mean score for the region chosen and divide the result by the respective standard deviation (values provided in Table S2, below):

$$Std\ Score_{Attitude\ to\ violence_i} = \frac{(Score_{Attitude\ to\ violence_i}) - (mean)}{standard\ deviation}$$

$$Std\ Score_{Social\ independence_i} = \frac{(Score_{Social\ independence_i}) - (mean)}{standard\ deviation}$$

$$Std\ Score_{Decision-making_i} = \frac{(Score_{Decision-making_i}) - (mean)}{standard\ deviation}$$

Table S1. Item weights used in the equations for estimating individual scores for each domain of the SWPER Index.

Item (v)	λ_{v1} Attitude to violence	λ_{v2} Social independence	λ_{v3} Decision-making
1. Beating not justified if wife goes out without telling husband	0.508	-0.012	-0.003
2. Beating not justified if wife neglects the children	0.508	-0.026	-0.040
3. Beating not justified if wife argues with husband	0.526	0.001	0.007
4. Beating not justified if wife refuses to have sex with husband	0.538	0.001	0.028
5. Beating not justified if wife burns the food	0.588	-0.015	-0.020
6. Frequency of reading newspaper or magazine	0.083	0.422	0.121
7. Woman education	0.016	0.081	0.022
8. Age of respondent at cohabitation	-0.006	0.133	-0.012
9. Age of respondent at first birth	-0.010	0.139	-0.016
10. Age difference: woman's minus husband's age	0.001	0.031	0.013
11. Education difference: woman's minus husband's years of schooling	0.002	0.054	0.001
12. Who usually decides on respondent's health care	0.001	-0.004	0.599
13. Who usually decides on large household purchases	-0.017	-0.022	0.601
14. Who usually decides on visits to family or relatives	0.002	-0.034	0.619

Table S2. Mean and standard deviation for the standardization of the SWPER scores.

	Attitude to violence		Social Independence		Decision-making	
**Region**	Mean	Std. dev.	Mean	Std. dev.	Mean	Std. dev.
South Asia	-0.138	1.804	-0.121	1.452	-0.097	1.546
East Asia & Pacific	0.238	1.563	0.757	1.550	0.792	0.950
Europe and Central Asia	0.256	1.701	1.286	1.169	0.619	1.296
Middle East & North Africa	-0.167	1.923	0.371	1.549	0.014	1.449
West & Central Africa	-0.601	2.030	-0.683	1.346	-0.913	1.562
Eastern & Southern Africa	0.094	1.745	-0.142	1.350	0.246	1.283
Latin America & Caribbean	1.084	0.852	0.460	1.546	0.674	1.049
SWPER Global	0.000	1.811	0.000	1.526	0.000	1.502

Table S3. Cut-offs used to categorize the SWPER domains into low, medium and high empowerment levels.

	Attitude to violence	Social independence	Decision-making
Low empowerment	≤-0.700	≤-0.559	≤-1.000
Medium empowerment	>-0.700 ≤0.400	>-0.559 ≤0.293	>-1.000 ≤0.600
High empowerment	>0.400	>0.293	>0.600

Figure S1. Distribution of the attitude to violence, social independence and decision making SWPER scores with the red lines indicating the cut-offs defined to categorize the empowerment levels in three groups (low, medium and high empowerment).

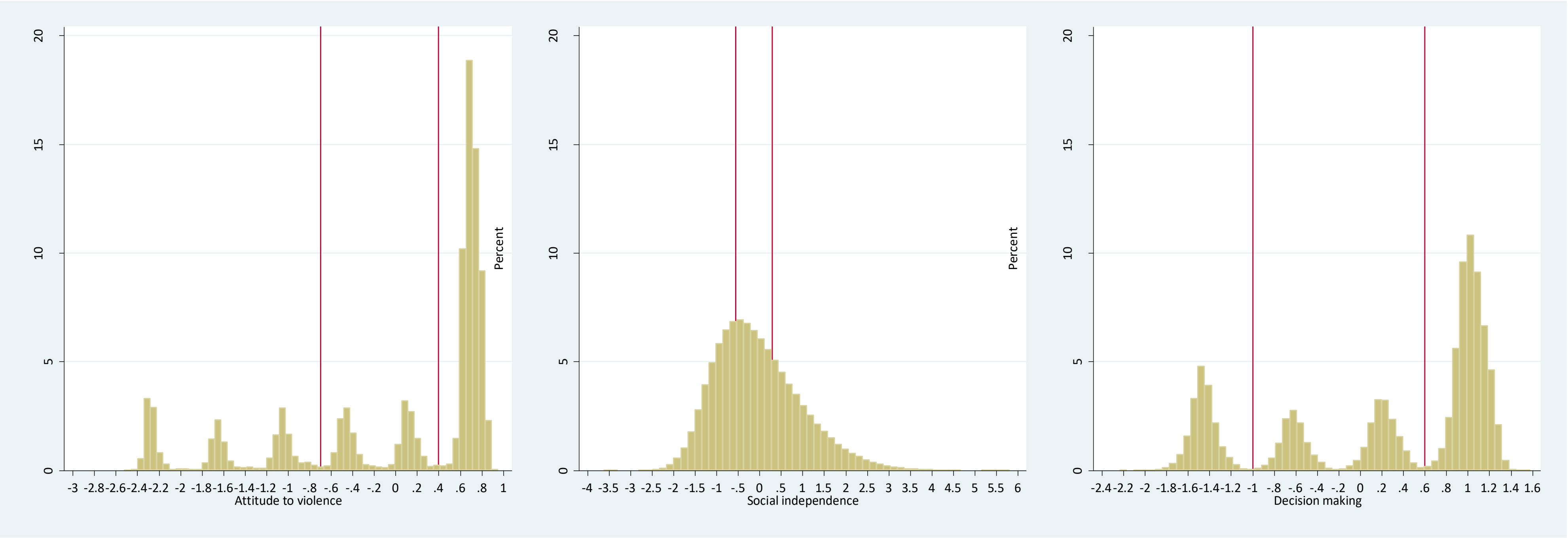

Table S4. Proportion of women aged 15-49 who are married or in a union for each country analysed.

Country	Year	Proportion of women married/in union
**South Asia**		
Afghanistan	2015	68.9*
Bangladesh	2014	81.6*
India	2015	73.1
Maldives	2009	68.6
Nepal	2016	76.8
Pakistan	2012	63.7*
**East Asia & the Pacific**		
Cambodia	2014	67.7
Indonesia	2012	73.4
Myanmar	2015	60.2
Philippines	2017	59.9
Timor-Leste	2016	61.1
**Europe & Central Asia**		
Albania	2008	65.9
Armenia	2015	63.7
Azerbaijan	2006	62.4
Kyrgyzstan	2012	64.0
Moldova	2005	66.4
Tajikistan	2012	67.4
Ukraine	2007	60.2
**Middle East & North Africa**		
Egypt	2014	69.8*
Morocco	2003	52.3
**West & Central Africa**		
Benin	2011	70.4
Burkina Faso	2010	79.4
Cameroon	2011	63.5
Chad	2014	74.8
Congo DR	2013	64.2
Cote d'Ivoire	2011	62.7
Gabon	2012	53.1
Gambia	2013	66.4
Ghana	2014	56.6
Guinea	2012	73.6
Liberia	2013	58.3
Mali	2012	84.6
Niger	2012	88.5
Nigeria	2013	71.5
Sao Tome & Principe	2008	65.7
Senegal	2017	64.9
Sierra Leone	2013	65.5
Togo	2013	66.3
**Eastern & Southern Africa**		
Angola	2015	55.3
Burundi	2016	56.6
Comoros	2012	61.2
Eswatini	2006	41.3
Ethiopia	2016	65.2
Kenya	2014	59.7
Lesotho	2014	54.6
Madagascar	2008	69.3
Malawi	2015	65.7
Mozambique	2011	67.9
Namibia	2013	34.0
Rwanda	2014	51.7
Tanzania	2015	61.9
Uganda	2016	60.6
Zambia	2013	60.1
Zimbabwe	2015	61.8
**Latin America & Caribbean**		
Bolivia	2008	60.0
Dominican Republic	2013	54.0
Guatemala	2014	58.0
Guyana	2009	58.4
Haiti	2016	51.5
Honduras	2011	56.5
Nicaragua	2001	56.8
Peru	2016	57.5

Note: *Data not available from DHS as these surveys only included ever-married women. Information from United Nations, Department of Economic and Social Affairs, Population Division (2020). Estimates and Projections of Women of Reproductive Age Who Are Married or in a Union: 2020 Revision. New York: United Nations.

Table S5. Coverage of low- and middle-income countries included in the development of the SWPER global.

Country	Included	Not included
**All (48%)**		
**South Asia (75%)**		
Afghanistan	X	
Bangladesh	X	
Bhutan		X
India	X	
Maldives	X	
Nepal	X	
Pakistan	X	
Sri Lanka		X
**East Asia & the Pacific (33%)**		
Cambodia	X	
China		X
Indonesia	X	
Korea DPR		X
Lao		X
Mongolia		X
Myanmar	X	
Papua New Guinea		X
Philippines	X	
Samoa		X
Solomon Islands		X
Thailand		X
Timor-Leste	X	
Vanuatu		X
Vietnam		X
**Europe & Central Asia (37%)**		
Albania	X	
Armenia	X	
Azerbaijan	X	
Belarus		X
Bosnia and Herzegovina		X
Georgia		X
Kazakhstan		X
Kosovo		X
Kyrgyzstan	X	
Macedonia		X
Moldova	X	
Montenegro		X
Serbia		X
Tajikistan	X	
Turkey		X
Turkmenistan		X
Ukraine	X	
Uzbekistan		X
Yugoslavia		X
**Middle East & North Africa (14%)**		
Algeria		X
Djibouti		X
Egypt	X	
Iraq		X
Jordan		X
Lebanon		X
Morocco	X	
Oman		X
Qatar		X
State of Palestine		X
Sudan		X
Syria		X
Tunisia		X
Yemen		X
**West & Central Africa (72%)**		
Benin	X	
Burkina Faso	X	
CAR		X
Cameroon	X	
Cape Verde		X
Chad	X	
Congo Brazzaville		X
Congo DR	X	
Cote d'Ivoire	X	
Equatorial Guinea		X
Gabon	X	
Gambia	X	

Ghana	X	
Guinea	X	
Guinea Bissau		X
Liberia	X	
Mali	X	
Mauritania		X
Niger	X	
Nigeria	X	
Sao Tome & Principe	X	
Senegal	X	
Sierra Leone	X	
Togo	X	
Zaire		X
**Eastern & Southern Africa (76%)**		
Angola	X	
Botswana		X
Burundi	X	
Comoros	X	
Eritrea		X
Eswatini	X	
Ethiopia	X	
Kenya	X	
Lesotho	X	
Madagascar	X	
Malawi	X	
Mozambique	X	
Namibia	X	
Rwanda	X	
Somalia		X
South Africa		X
South Sudan		X
Tanzania	X	
Uganda	X	
Zambia	X	
Zimbabwe	X	
**Latin America & Caribbean (30%)**		
Argentina		X
Barbados		X
Belize		X
Bolivia	X	
Brazil		X
Colombia		X
Costa Rica		X
Cuba		X
Dominican Republic	X	
Ecuador		X
El Salvador		X
Guatemala	X	
Guyana	X	
Haiti	X	
Honduras	X	
Jamaica		X
Mexico		X
Nicaragua	X	
Panama		X
Paraguay		X
Peru	X	
Puerto Rico		X
St Lucia		X
Suriname		X
Trinidad and Tobago		X
Uruguay		X
Venezuela		X
